# Development and validation of a nomogram for assessing survival in patients with hepatocellular carcinoma after hepatectomy

**DOI:** 10.1042/BSR20192690

**Published:** 2020-06-16

**Authors:** Rong-Rui Huo, Xu Liu, Jing Cui, Liang Ma, Kun-Hua Huang, Cai-Yi He, Yang Yang, Xue-Mei You, Wei-Ping Yuan, Bang-De Xiang, Jian-Hong Zhong, Le-Qun Li

**Affiliations:** 1 Hepatobiliary Surgery Department, Guangxi Liver Cancer Diagnosis and Treatment Engineering and Technology Research Center, Guangxi Medical University Cancer Hospital, Nanning 530021, China; 2 Editorial Office of Guangxi Medical University Cancer Hospital, Nanning 530021, China; 3 Grade 2016, Basic Medical College of Guangxi Medical University, Nanning 530021, China; 4 Chemotherapy Department, The First Affiliated Hospital of Guangxi Medical University, Nanning 530021, China

**Keywords:** Hepatectomy, Hepatocellular Carcinoma, Nomogram, Survival

## Abstract

**Background and aim:** Assessing the average survival rate of patients with hepatocellular carcinoma (HCC) after hepatectomy is important for making critical decisions in everyday clinical practice. The present study aims to develop and validate a nomogram for assessing the overall survival probability for such patients.

**Methods:** The putative prognostic indicators for constructing the nomogram were identified using multivariable Cox regression and model selection based on the Akaike information criterion. The nomogram was subjected to internal and external validation. The nomogram endpoints were death within 1, 3, and 5 years.

**Results:** A consecutive sample of 522 HCC patients who underwent potentially curative hepatectomy was retrospectively analyzed. Age, Barcelona clinic liver cancer (BCLC) stage, tumor size, alanine transaminase, alpha fetal protein, and serum prealbumin were included in the final model. The nomogram's discriminative ability was good in the training set (C-index was 0.74 for 1 year, 0.73 for 3 years, 0.70 for 5 years) and was validated using both an internal bootstrap method (C-index was 0.73 for 1 year, 0.72 for 3 years, 0.69 for 5 years) and an external validating set (C-index was 0.72 for 1 year, 0.72 for 3 years, 0.69 for 5 years). The calibration plots for the endpoints showed optimal agreement between the nomogram's assessment and actual observations.

**Conclusions:** The nomogram (an Excel-based tool) can be useful for assessing the probability of survival at 1, 3, and 5 years in patients with HCC after hepatectomy.

## Introduction

Hepatocellular carcinoma (HCC) is a common malignant neoplasm and one of the leading causes of cancer-related death [1]. Hepatectomy is one of the main radical treatments for HCC. However, its 5-year recurrence rate is up to 70% [2], and the 5-year overall survival (OS) is only 37% for patients with portal hypertension, 30% for those with multiple tumors, and 18% for those involving macrovascular invasion [3]. Accurately assessing the average survival rate of this population is important for making critical decisions in everyday clinical practice, and how to register for hospice care design. However, assessing the life expectancy of HCC patients after hepatectomy is a challenge for hepatologist because of the heterogeneity of patients and the background liver disease.

In recent years, nomograms have been widely accepted in the oncology community as reliable tools for assessing patients’ prognoses. Nomograms are graphical depictions of predictive statistical models for individual patients [4], and they have been developed for various types of cancers [5–7]. Because the use of nomograms has a demonstrated advantage over the traditional staging systems used to predict patient outcomes for many cancers, nomograms have been proposed as an alternative method or even as a new standard to guide treatment allocation for cancer patients [8]. In the present study, we aimed to develop and validate a nomogram for assessing the survival probability at 1, 3, and 5 years in patients with HCC after hepatectomy.

## Methods

### Patients

**Figure 1:**
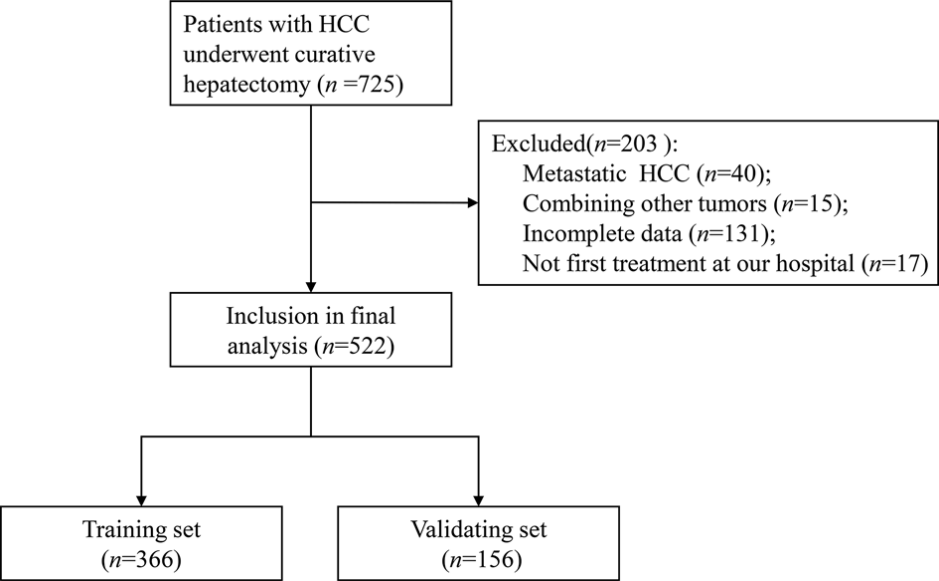
Numbers of patients enrolled and outcomes in the training set and validating set

Data from HCC patients who underwent hepatectomy at the Guangxi Medical University Cancer Hospital between August 2007 and August 2010 were used to develop the nomogram. HCC was histopathologically confirmed. Patients who underwent other treatments for HCC prior to hepatectomy or with other malignancies were excluded. Patients were also excluded if they had presented distant extra-hepatic metastases or had incomplete information. The process for selecting patients for model development is presented in Figure 1. The present study was approved by the Ethics Commitment of the Guangxi Medical University Cancer Hospital. All included participants provided written informed consent before their data were analyzed.

### Study design and data collection

The present study is reported in accordance with the Transparent Reporting of a Multivariable Prediction Model for Individual Prognosis or Diagnosis (TRIPOD) reporting guideline statement checklist for prediction model development and validation [9]. The nomogram endpoints were death within 1, 3, and 5 years from the date of the first hepatectomy. Patients were randomly divided into training set and validating set according to the ratio of 7:3. The presumptive nomogram prognosticators were clinical and pathological parameters selected a priori that correlated with prognoses of patients with HCC, including age, sex, liver function categorized according to Child–Pugh Category criteria, Barcelona clinic liver cancer stage (BCLC stage), serum prealbumin, hepatitis B surface antigen (HBsAg), albumin (ALB), aspartate aminotransferase (AST), alanine aminotransferase (ALT), total bilirubin (TBIL), alpha-fetoprotein (AFP), and pathology of HCC involving cirrhosis, tumor number and tumor size, integrity of tumor capsule, and presence or absence of macrovascular invasion. Mircrovascular invasion was not reported for most of the included patients. Therefore, the microvascular invasion was not included in the analysis. All data (except pathology variables) were retrospectively collected from electronic medical records, and all measurements were obtained before initiating date of surgery. In order to ensure the accuracy and reliability of data in the present study, measures to construct stander quality control were planned and performed, such as table for fulfillment of information form patients and code for replacement of categorical data were made before the start of study; two researchers independently and blindly constructed date collection and organization, which must be correct and consistent, otherwise led to rechecked out date procedure.

### Follow-up

All patients had been traced from the day of hepatectomy to August 31, 2018, until the patient dies or lost to follow-up. Most routine patient follow-up appointments including recurrence of HCC, time of recurrence, survival status, data of death, and liver function were supplied by phone call to patients or data management system of our hospital. Follow-up began from the first month after hepatectomy. Survival time was defined as the interval (in months) from the date of hepatectomy to the date of last follow-up (August 31, 2018) or death.

### Statistical analysis

Categorical variables were presented as numbers and percentages, and continuous variables were presented as the mean and standard deviation (SD). Survival curves were depicted using the Kaplan–Meier method. Multivariable Cox regression analysis was performed to calculate the hazard ratios (HRs) and 95% confidence intervals (CIs) of the putative prognosticators and build the nomogram. Categorical prognostic indicators (sex, BCLC stage, Child–Pugh, macrovascular invasion, tumor capsule, liver cirrhosis, and HBsAg) were classified based on clinical findings and were entered as dummy variables. Continuous prognostic indicators (age, tumor size, tumor number, ALB, AST, ALT, and TBIL) were transformed into categorical variables based on routine cutoff points in the clinical application. Serum prealbumin was entered linearly or modeled using restricted cubic splines. Restricted cubic splines assume that the effect of the prognostic indicator on the outcome is a smooth piecewise cubic polynomial with linear trails. They provide flexibility in fitting highly curved relationships, avoid heavy influence from outlying prognostic indicators, and may provide better power than dichotomizing continuous variables [10]. A greater number of knots implies additional flexibility of the fitted curve. In the present study, the positions of the knots were set as the 25th, 50th, and 75th percentiles with three knots. For each setting combination for continuous variables, we generated a candidate model using backward elimination with Akaike information criterion (AIC) as the selection criterion [11]. We selected the model with the lowest AIC as the final prediction model. The performance of the model was evaluated with calibration plots (which determine the agreement between the observed and estimated survival probability using the Kaplan–Meier method) and validation indices (which determine the discriminative ability of the models). Bias-corrected internal calibration was performed at 1, 3, and 5 years using 500 bootstraps resamples. For internal validation, C-index at 1, 3, and 5 years was calculated to represent discriminative ability [12]. A C-index significantly greater than 0.5 indicates good discrimination of the model [12]. Bias-corrected internal validation was also performed by evaluating C-index under 500 bootstrap resamples. External calibration and validation were performed using the same methods on the validating data set. Based on the fitted prediction model, we produced a corresponding nomogram and an interactive Excel-based survival probability application, version 0.10. Statistical analyses were performed using R version 3.5.1 software (http://www.r-project.org/), extension packages, including ‘survival’, ‘rms’, and ‘survminer’ were also used.

## Results

### Training set

**Figure 2:**
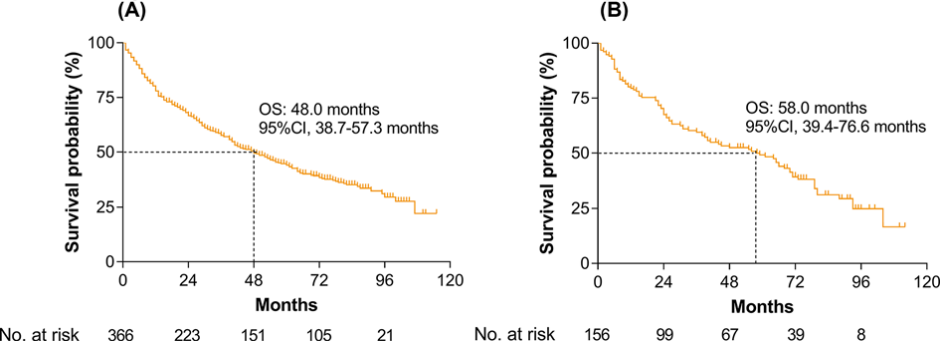
Kaplan–Meier curves for overall survival (**A**) In the training set and (**B**) in the validating set.

The training set included 366 eligible patients whose clinical and pathological characteristics were shown in Table 1. Of the 366 patients in the training set, 315 patients (86.1%) were male, and the mean (SD) age was 48.5 (11.3) years. After a median follow-up of 56.1 months, there were 77 (21.0%), 144 (39.3%), and 185 (50.5%) deaths within 1, 3, and 5 years, respectively. The median OS was 48.0 months (95%CI, 38.7–57.3 months). The Kaplan–Meier curve is shown in Figure 2A.

**Table 1:** Clinicopathological characteristics of the patients

Baseline characteristics	Training set (*n* = 366)	Validating set (*n* = 156)	*P* value*
Age (year)	48.54 (11.31)	48.69 (11.24)	0.891
≤60	315 (86.1%)	135 (86.5%)	
>60	51 (13.9%)	21 (13.5%)	
Serum prealbumin (mg/l)	204.59 (66.85)	197.41 (57.47)	0.243
Gender			0.808
Female	315 (86.1%)	133 (85.3%)	
Male	51 (13.9%)	23 (14.7%)	
Child–Pugh			0.735
A	347 (94.8%)	149 (95.5%)	
B	19 (5.2%)	7 (4.5%)	
BCLC			0.169
0/A	205 (56.0%)	93 (59.6%)	
B	71 (19.4%)	36 (23.1%)	
C	90 (24.6%)	27 (17.3%)	
Macrovascular invasion			0.068
No	276 (75.4%)	129 (82.7%)	
Yes	90 (24.6%)	27 (17.3%)	
Tumor number			0.507
>3	321 (87.7%)	140 (89.7%)	
≤3	45 (12.3%)	16 (10.3%)	
Tumor size (cm)			0.635
>5	149 (40.7%)	67 (42.9%)	
≤5	217 (59.3%)	89 (57.1%)	
Tumor capsule			0.175
Complete	171 (46.7%)	83 (53.2%)	
Incomplete	195 (53.3%)	73 (46.8%)	
HBsAg			0.462
−	56 (15.3%)	20 (12.8%)	
+	310 (84.7%)	136 (87.2%)	
Liver cirrhosis			0.186
No	105 (28.7%)	36 (23.1%)	
Yes	261 (71.3%)	120 (76.9%)	
AFP (ng/ml)			0.756
≤400	227 (62.0%)	99 (63.5%)	
>400	139 (38.0%)	57 (36.5%)	
ALB (g/l)			0.325
≤35	41 (11.2%)	13 (8.3%)	
>35	325 (88.8%)	143 (91.7%)	
AST (U/l)			0.140
≤40	183 (50.0%)	67 (42.9%)	
>40	183 (50.0%)	89 (57.1%)	
ALT (U/l)			0.940
≤40	189 (51.6%)	80 (51.3%)	
>40	177 (48.4%)	76 (48.7%)	
TBIL (μmol/l)			0.651
≤21	321 (87.7%)	139 (89.1%)	
>21	45 (12.3%)	17 (10.9%)	

Data are *n* (%) or mean (SD), unless otherwise specified.

* Pearson's chi-squared test or Fisher's exact test. *P* value<0.05 indicates a significant difference between the two groups.

**Figure 3:**
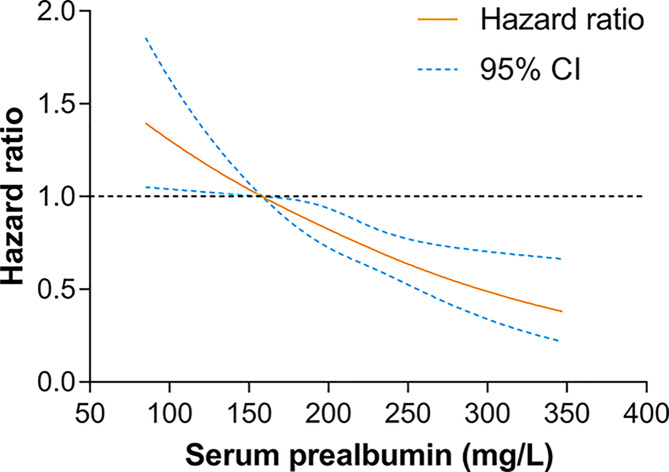
Dose–response relationships between serum prealbumin and the risk of mortality

**Figure 4:**
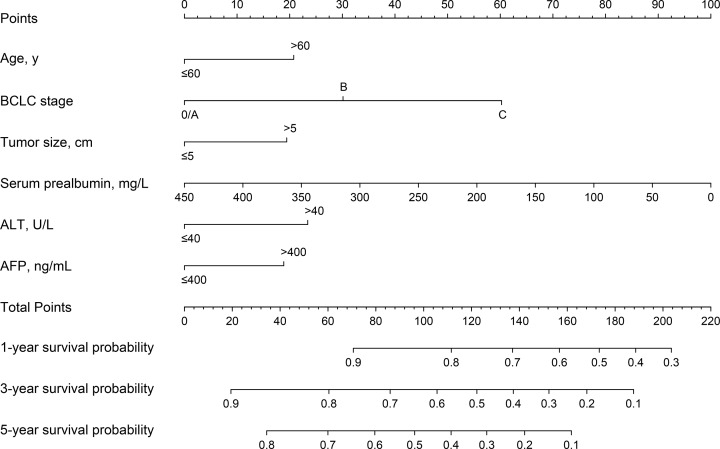
The nomogram developed in the present study A nomogram to predict the survival time of patients with HCC after hepatectomy. The nomogram allows the user to obtain the probability of 1-, 3-, and 5-year survival probability corresponding to a patient's combination of covariates. As an example, locate the patient's BCLC stage and draw a line straight upward to the ‘Points’ axis to determine the score associated with that BCLC stage. Repeat the process for each variable, and sum the scores achieved for each covariate, and locate this sum on the ‘Total Points’ axis. Draw a line straight down to determine the likelihood of 1-, 3-, or 5-year survival probability.

Univariable analysis results were shown in Table 2. We then evaluated the potential dose–response relationships between serum prealbumin and the risk of death using restricted cubic spline models (Figure 3). A linear dose–response relationship was observed (linear, *P*<0.001). Therefore, serum prealbumin was entered into the Cox model as continuous variables. After backward elimination and model selection based on AIC, six prognostic indicators, including age (>60 vs. ≤60 year: HR, 1.48; 95%CI, 1.02–2.15), BCLC stage (B vs. 0/A: HR, 1.50; 95%CI, 1.05–2.14; C vs. 0/A: HR, 3.08; 95%CI, 2.22–4.28), tumor size (>5 vs. ≤5 cm: HR, 1.42; 95%CI, 1.05–1.91), serum prealbumin (HR, 0.99; 95%CI, 0.98–1.00), ALT (>40 vs. ≤40 U/L: HR, 1.54; 95%CI, 1.17–2.03), and AFP (>400 vs. ≤400 ng/mL: HR, 1.41; 95%CI, 1.06–1.88) were included in the final model (AIC = 2189.3) for the construction of the nomogram. Results of the final multivariable Cox regression model were shown in Table 3. The nomogram, for survival probabilities in this particular population, was shown in Figure 4. By summing the points from each variable, locating the total points on the scale, and drawing a straight line down to the endpoint scales, the nomogram allows users to easily estimate the probability of survival at 1, 3, and 5 years.

**Table 2:** Univariable analysis of putative clinicopathological variables

Baseline characteristics	Hazard ratio (95% CI)	*P* value
Age (>60 years vs. ≤60 years)	1.21 (0.84–1.74)	0.296
Serum prealbumin (mg/l)	0.99 (0.98–1.00)	<0.001
Gender (male vs. female)	1.15 (0.79–1.68)	0.470
Child–Pugh (B vs. A)	0.86 (0.44–1.68)	0.664
BCLC stage		
B vs. 0/A	1.62 (1.14–2.32)	0.008
C vs. 0/A	3.19 (2.33–4.38)	<0.001
Macrovascular invasion (yes vs. no)	2.66 (1.98–3.58)	<0.001
Tumor number (>3 vs. ≤3)	1.94 (1.32–2.85)	0.001
Tumor size (>5 cm vs. ≤5 cm)	1.87 (1.41–2.50)	<0.001
Tumor capsule (complete vs. incomplete)	1.84 (1.40–2.42)	<0.001
HBsAg (+ vs. −)	1.21 (0.82–1.77)	0.333
Liver cirrhosis (yes vs. no)	0.87 (0.65–1.16)	0.333
AFP (>400 ng/ml vs. ≤400 ng/ml)	1.56 (1.19–2.05)	0.001
ALB (>35 g/l vs. ≤35 g/l)	0.82 (0.54–1.24)	0.339
AST (>40 U/L vs. ≤40 U/l)	1.67 (1.27–2.19)	<0.001
ALT (>40 U/L vs. ≤40 U/l)	1.53 (1.17–2.00)	0.002
TBIL (>21 μmol/l vs. ≤21 μmol/l)	1.12 (0.75–1.68)	0.571

**Table 3:** Final model of the multivariable cox regression analysis

Baseline characteristics	Hazard ratio (95% CI)	*P* value
Serum prealbumin (mg/l)	0.99 (0.98–1.00)	<0.001
Age (>60 years vs. ≤60 years)	1.48 (1.02–2.15)	0.041
BCLC stage		
B vs. 0/A	1.50 (1.05–2.14)	0.027
C vs. 0/A	3.08 (2.22–4.28)	<0.001
Tumor size (>5 cm vs. ≤5 cm)	1.42 (1.05–1.91)	0.022
AFP (>400 ng/ml vs. ≤400 ng/ml)	1.41 (1.06–1.88)	0.017
ALT(>40 U/l vs. ≤40 U/l)	1.54 (1.17–2.03)	0.002

**Figure 5:**
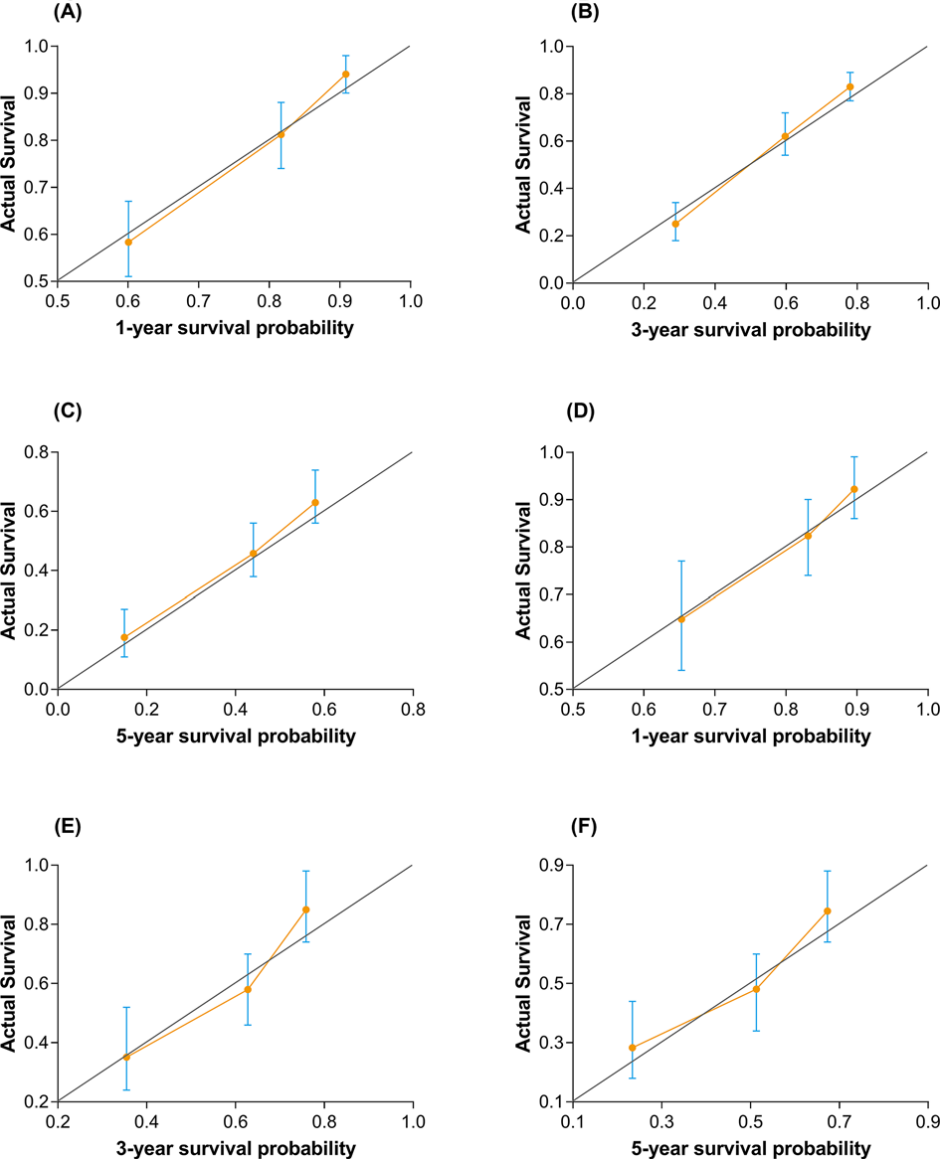
Calibration plots for estimating survival probability at 1, 3, and 5 years Calibration plots are shown for the training set (**A**–**C**) and the external validating set (**D**–**F**). The 45° gray line is the reference line that indicates where a perfect calibration would lie.

**Figure 6:**
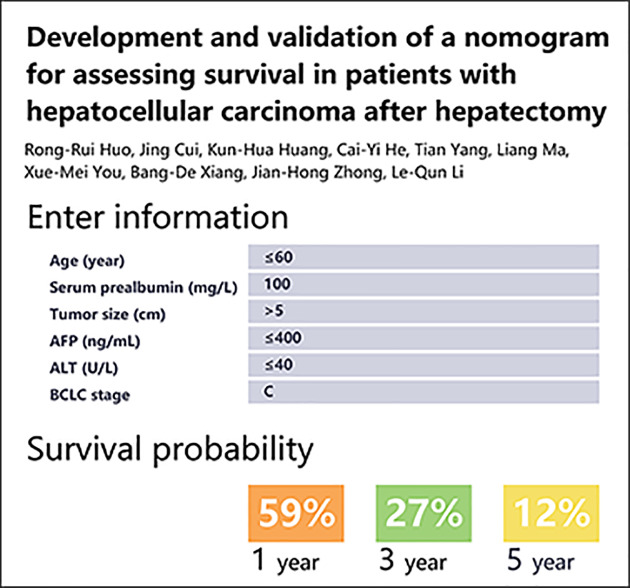
The excel-base tool base on the nomogram

The calibration plot for internal validation showed excellent agreement of 1-, 3-, and 5-year survival probabilities between the nomogram's estimated and actual observations (Figure 5A–C). All the predicted lines were overlapped well with the reference line, demonstrating the good performance of the nomogram. In terms of discriminative ability, C-index on the training set was 0.74 (95%CI, 0.70–0.78) for 1 year, 0.73 (95%CI, 0.68–0.79) for 3 years, and 0.70 (95%CI, 0.65–0.75) for 5 years. Bootstrap validation showed that the optimism corrected C-index was 0.73 (95%CI, 0.69–0.77) for 1 year, 0.72 (95%CI, 0.67–0.76) for 3 years, and 0.69 (95%CI, 0.68–0.74) for 5 years, which indicated good discrimination. An excel-base tool for the convenience of clinicians was also developed (Figure 6).

### Validating set

The validating set included 156 eligible patients whose clinicopathological characteristics were summarized in Table 1. Of the 156 patients in the validating set, 133 patients (85.3%) were male, and the mean (SD) age was 48.69 (11.24) years. After a median follow-up of 56.1 months, there were 31 (19.9%), 59 (37.8%), and 72 (46.2%) deaths within 1, 3, and 5 years, respectively. The median survival time was 58.0 months (95%CI, 39.4–76.6 months). The Kaplan–Meier curve was shown in Figure 2B.

The calibration plot for external validation (Figure 5D–F) showed good agreement between the nomogram's estimated and the observed probabilities of survival at 1, 3, and 5 years. The predicted line overlapped well with the reference line, demonstrating the good performance of the nomogram. C-index for the established nomogram in the validating set was 0.72 (95%CI, 0.65–0.78) for 1 year, 0.72 (95%CI, 0.66–0.79) for 3 years, and 0.69 (95%CI, 0.65–0.75) for 5 years, which showed that this nomogram remains valid for use in an external set.

## Discussion

There are several major findings in the present study. First, in this training cohort, hepatectomy resulted in the death of 21.0% at 1 year, 39.3% at 3 years, and 50.5% at 5 years. These results are generally consistent with previous studies and confirm that death is a frequent event in patients with HCC after hepatectomy [13]. Second, the serum prealbumin is closely related to the prognosis of patients with hepatectomy, indicating this simple biomarker could provide an important prognostic reference for HCC patients. Third, we have constructed and validated a nomogram that can be used for assessing the survival probability of patients with HCC after hepatectomy. This information is crucial for hepatologist in treatment decision making and engaging patients in end-of-life discussions and/or hospice referrals appropriate times.

The attending physician plays an important role in the treatment of HCC patients. In addition to judging whether a patient needs surgery according to clinical guidelines, it is often necessary to consider the patient's prognosis and the patient's wishes. However, most doctors lack confidence in estimating patient life expectancy [14], making them more likely to reject patient prognosis information and miss out on the best time to conduct end-of-life discussions and/or provide hospice referrals [15]. In addition, studies have shown that many patients want to know the average survival rate after liver resection [16]. Therefore, the present study selected three clinically valuable research endpoints (1, 3, and 5 years). Our nomogram can not only assist clinicians in making critical treatment decisions but also provide patients with very important survival information.

In the present study, We have further analyzed the independent factors related to the prognosis of hepatectomy patients. In accordance with published studies [17–19], our study showed that the age (>60 years), tumor size (>5 cm), AFP level (>400 ng/ml), ALT level (>40 U/l), and BCLC stage were linked with higher risk of death after hepatectomy. Notably, serum prealbumin, a plasma protein synthesized by hepatocytes, is an indicator of nutritional status in cancer patients. The present study demonstrated a linear dose–response relationship between serum prealbumin levels and the prognosis of HCC after hepatectomy, suggesting that higher levels of prealbumin may reduce the risk of death in HCC patients [20]. Altogether, the extent of tumor involvement, as reflected by these factors, heavily determines the risk of death.

Base on the Cox model, we developed an assessable nomogram from a prognostic cohort undergoing hepatectomy. The nomogram was employed to generate an easy-to-apply model that can be used for individualized risk estimation for OS after hepatectomy. The advantage of using this nomogram is that all variables are common clinical features and do not require advanced mathematical calculations. We had also developed an excel-base tool for the convenience of clinicians. Earlier studies that used the prognostic nomogram in patients with HCC after hepatectomy did not incorporate the serum prealbumin into the model [21–22]. Importantly, serum prealbumin is completely produced by liver cells and is not interfered by venous supplementation. It has the ability to evaluate the status of liver cells and can be used as one of the potential indicators to predict the risk of death or prognosis of patients [18,23]. In the restricted cubic splines model and the multivariate Cox model, the risk of death was reduced by 1% for every additional unit of serum prealbumin, and both showed a linear dose-response relationship, indicating serum prealbumin deficiency is associated with poor prognosis. However, the specific mechanism by which serum prealbumin affects the prognosis of HCC patients remains unclear.

During a median follow-up duration of 56.1 months, 58.5% patients had died in the training set; this number is 35 times the number of predictors used in the nomogram. This feature indicates that the Cox regression model has sufficient statistical power to predict survival probability [24]. Based on the independent prognostic factors, the nomograms established in the present study performed well in predicting survival. The C-index of the training set and the validation set at 1, 3, and 5 years were both higher than 0.70. Notably, the calibration plots for OS at 1, 3, and 5 years well matched the idealized 45° line, proving that the nomogram has higher prediction accuracy. Combining the results of C-index and calibration plot, internal and external validation results demonstrate the high performance of the nomogram. This nomogram model can be considered a feasible tool to predict the probability of survival at 1, 3, and 5 years in patients with HCC undergoing hepatectomy.

The present study has several limitations. First, the present study was retrospectively designed, and thus, incomplete adherence to the post-resection follow-up protocol and potential confounders for OS were inevitable. Second, this was a single-center study that cannot avoid case selection bias. Thus a robust nomogram should be validated externally in different cohorts. Third, the nomogram was generated from patients who had not presented distant extra-hepatic metastases, it may not be suitable for patients with metastases. This limit the use of our nomogram. Last, externally validated data comes from the cohort, and this approach may have a risk of overfitting. So external validation is essential from different research centers to prove the clinical utility of the models.

## Conclusions

We developed and validated a nomogram to estimate the probability of survival at 1, 3, and 5 years in patients with HCC after hepatectomy. Based on six objective and easy-to-acquire variables, this nomogram and Excel-based tool provided clinically relevant information to the patient and can assist hepatologist in treatment decision making and engaing patients in end-of-life dicussions and/or hospice referrals appropriate times.

## Abbreviations

AFP: alpha-fetoprotein
AIC: Akaike information criterion
ALB: albumin
ALT: alanine aminotransferase
AST: aspartate aminotransferase
BCLC: Barcelona clinic liver cancer
CI: 95% confidence interval
HBsAg: hepatitis B surface antigen
HCC: hepatocellular carcinoma
HR: hazard ratio
OS: overall survival
TBIL: total bilirubin
